# Getting by With a Little Help From Fungal Friends: Seed‐Associated Endophytes Contribute to Seed Defense Against Fungal Pathogens in Keystone Tropical Trees

**DOI:** 10.1002/ece3.74184

**Published:** 2026-08-12

**Authors:** Sumashini Sundararajan, Gauranshi Chamoli, James W. Dalling, Meghna Krishnadas

**Affiliations:** ^1^ Department of Plant Biology University of Illinois at Urbana‐Champaign Champaign Illinois USA; ^2^ Nature Conservation Foundation Mysore India; ^3^ Smithsonian Tropical Research Institute Panama City Republic of Panama; ^4^ CSIR‐Centre for Cellular and Molecular Biology Hyderabad India; ^5^ Academy of Scientific and Innovative Research (AcSIR) Ghaziabad India; ^6^ TIFR National Center for Biological Sciences Bengaluru India

**Keywords:** beneficial fungi, *Ficus*, priority effects, seed persistence, seed‐fungal interactions

## Abstract

Soil‐borne fungal pathogens are known to be major causes of seed mortality in tropical forests; however, not all fungal infections of seeds are necessarily lethal. It has been hypothesized that seed‐associated fungi may contribute to seed defense and persistence in soil seed banks and are especially important for non‐dormant seeds with limited physical and chemical defenses. This study examined microbially mediated seed defense in two fig (*Ficus*) species from a tropical forest in the Western Ghats, India. One species, *Ficus callosa* has a free‐standing habit (seeds germinate in the soil), and the other, *Ficus beddomei* is a hemi‐epiphyte (seeds germinate in the canopy of host trees and send roots to soil). We predicted that (i) fungi infecting seeds prior to soil exposure will have less negative effects on seed survival compared to soil‐borne fungi (ii) the hemi‐epiphytic figs are more susceptible to soil‐borne fungi, and (iii) prior infection by non‐pathogenic, endophytic fungi excludes pathogens and increases seed survival. We inoculated seeds using seed‐associated fungal isolates obtained from the two fig species prior to and after exposing seeds to soil, classified their effects as endophytic or pathogenic, then tested the effect of pre‐treatment of seeds with endophytes before exposure to putative pathogens. We found that inoculation with fungal taxa isolated from seeds prior to soil exposure did not affect seed germination for either fig species, while soil‐borne fungi were frequently pathogenic, especially in the hemi‐epiphytic fig. Incubation with endophytic fungi prior to pathogen exposure increased seed survival depending on endophyte and pathogen identities, with stronger effects in the free‐standing fig. *Synthesis*: Our findings show that prior seed infection by seed‐associated endophytes could be a plausible mechanism of seed defense against fungal pathogens. It highlights the potential for dynamic seed‐fungal interactions to increase seed survival, with implications for tree populations and diversity in tropical forests.

## Graphical abstract

This study explored microbially mediated seed defense in fig (Ficus) species through inoculation experiments. We show that priority effects from endophytic fungi contribute to increased seed survival in the presence of fungal pathogens.

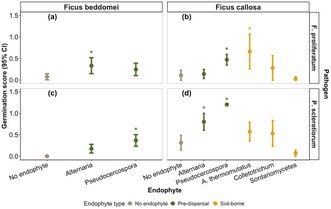

## 1 Introduction

Microbes play a crucial role in shaping plant diversity via interactions spanning across mutualism, parasitism and saprotrophy (Lutzoni et al. 2018; Delaux and Schornack 2021). The plant microbiome is also increasingly viewed as a key determinant of plant health (Trivedi et al. 2020), shaped by interactions between the host and the environment (Leveau 2024). In addition to affecting germination, growth, nutrient acquisition and abiotic stress tolerance (Richardson and Simpson 2011; Trivedi et al. 2022; Chieb and Gachomo 2023), the plant microbiome can also confer resistance against herbivores and pathogens (Pieterse and Van Wees 2015; Berg et al. 2017). While disease resistance has been mostly studied in roots and leaves, microbially mediated defenses may also contribute to seed survival and persistence, especially in microbially‐rich soils in tropical forests (Dalling et al. 2020). Seeds represent a crucial stage in the life cycle of plants, determining dispersal and establishment patterns. Seeds and young seedlings are especially vulnerable to attack from fungal and oomycete pathogens, resulting in substantial mortality in tropical forests (Dalling et al. 1998; Bell et al. 2006; Bagchi et al. 2014), and seed‐fungal interactions directly impact plant population dynamics and species coexistence (Terborgh 2012; Krishnadas et al. 2018; Viswanathan et al. 2019).

The ability to evade fungal pathogens in part depends on seed defense strategies. Seeds are some of the most heavily defended plant tissues (Zangerl and Bazzaz 1992; Chen and Moles 2018), employing a suite of physical structures and chemical compounds to defend themselves (Gong et al. 2015; Gripenberg et al. 2018). Seed defense characteristics are also linked to dormancy (Zalamea et al. 2018), where physical barriers or chemical inhibitors prevent germination until favorable environmental conditions are encountered (Baskin and Baskin 2000). While dormancy often relates to seed persistence in the soil, seeds of some pioneer species are capable of surviving for a year or longer in a non‐dormant state (Alvarez‐Buylla and Martínez‐Ramos 1990; Dalling et al. 1997; Dalling and Brown 2009). These ‘quiescent’ species await an appropriate environmental cue triggering germination. Quiescent seeds have relatively few chemical defenses (Zalamea et al. 2018) and are rapidly colonized by diverse communities of fungi in the soil (Sarmiento et al. 2017). It has been hypothesized that many quiescent species may rely on recruiting beneficial fungi to defend themselves (Dalling et al. 2011, 2020).

Seed‐associated fungal communities are predominantly composed of filamentous Ascomycota in the classes Sordariomycetes and Dothideomycetes (Nelson 2018; Zalamea et al. 2021; Simonin et al. 2022). Seed fungi can colonize seeds either during development on the maternal plant via vertical or horizontal transmission prior to seed dispersal (referred to as pre‐dispersal fungi in this study), or via horizontal transmission after seeds contact the soil (referred to as soil‐borne fungi). Therefore, many of the seed‐associated fungal genera are also found in soil and some in leaves (Arnold 2007; U'Ren et al. 2009). Some of these fungi are pathogenic, while others occur asymptomatically as endophytes (Nelson 2018). Since most studies including our study rely on surface‐sterilization alone to characterize seed fungi, these fungi are designated as seed‐associated endophytes rather than true seed endophytes (Nelson 2018) but referred to as endophytes henceforth for simplicity. The frequency of fungal colonization of seeds at the pre‐dispersal stage has been found to be quite low, with most fungal colonization occurring in the soil (Sarmiento et al. 2017). While endophytic fungi such as *Epichloë* in grasses may be transferred via seeds from plant to offspring (Saikkonen et al. 2010; Perez et al. 2017), such vertical transmission has not been commonly reported in tropical trees.

The role of endophytic fungi in disease resistance has received considerable attention at the seedling and adult plant stages. Endophytes may exclude pathogens through priority effects, where initial colonization by a fungus influences microbiome composition (Leopold and Busby 2020) or by altering the host response to a pathogen through the production of anti‐microbial compounds, modulation of plant hormone levels or induction of immune responses (Singh and Mathur 2004; Herre et al. 2007). In grasses, vertically transmitted endophytic fungi have been shown to offer systemic resistance to fungal pathogens at the adult plant stage (Saikkonen et al. 2010, 2016; Perez et al. 2017). Horizontally transmitted foliar endophytes can have a similar effect on disease incidence and severity in tropical trees (Arnold et al. 2003; Aponte Rolón et al. 2025).

However, it is unclear whether seed‐associated endophytes can defend seeds against pathogens and contribute to seed persistence in natural systems (Rojas et al. 2020; Kim et al. 2022). While high rates of seed mortality due to soil fungi are reported in tropical forests (Gallery et al. 2007), seed burial experiments have consistently recovered viable seeds with fungal infection even after burial for a year or more in soil (Sarmiento et al. 2017; Zalamea et al. 2021). In addition, naturally occurring seeds in soil that remain viable for over a decade have been shown to retain fungi (Zalamea et al. 2025). Furthermore, the presence of fungal endophytes has been shown to result in lower richness and diversity of seed‐borne microbiota (Liang et al. 2023) hinting at potential priority effects due to endophytic fungi.

Two key features of seed‐associated fungi suggest a dual role both as pathogens and in seed defense. First, using inoculation experiments Sarmiento et al. (2017) showed that fungi previously isolated from pioneer seeds can induce asymptomatic endophytic infection in one host and pathogenic infection in another, giving rise to “functional specificity” in seed‐fungal interactions. Host generalist seed‐associated fungi could, therefore, play a role in mediating density/distance dependent mortality and thus species diversity in tropical forests (Connell 1971; Janzen 1971; Comita et al. 2014). Second, culture‐based methods and metabarcoding show that while seed‐infecting fungal communities are diverse, individual seeds are typically infected by one or a very small number of fungi (Sarmiento et al. 2017, Ridout et al. 2019; Zalamea et al., unpublished data). Lower fungal diversity in seeds has been attributed to strong defense allocation to seeds and to exclusionary interactions among fungi (Newcombe et al. 2018). Consequently, the few fungal colonists in seeds may have strong effects on seeds and seedlings depending on their identity and activity as predicted by the “Primary Symbionts Hypothesis” (Newcombe et al. 2018). Here we explore whether primary colonization by endophytic fungi influences subsequent interactions with fungal pathogens with implications for seed survival, and therefore tree populations and forest diversity.

We used inoculation experiments to test (1) the pathogenicity of pre‐dispersal and soil‐borne fungi isolated from seeds, and (2) whether seed‐associated endophytes can protect seeds against fungal pathogens. We chose figs (*Ficus* spp., Moraceae), keystone tree species in tropical forests, as our model system. Fig seeds have high rates of infection in the soil by fungi with both endophytic and pathogenic lifestyles (Sarmiento et al. 2017). Figs also exhibit different habits that could impact their interactions with soil‐borne fungi. Specifically, we contrasted a free‐standing fig, *Ficus callosa*, that recruits from seeds that germinate from the soil seed bank, with a hemi‐epiphytic fig, *Ficus beddomei*, that germinates in the forest canopy and sends roots to the soil.

We predicted that (1) fungi acquired in the soil will have stronger negative effects on seed survival than pre‐dispersal fungi, as co‐evolutionary processes may result in asymptomatic, endophytic associations of pre‐dispersal fungi with potential fitness benefits to plants. (2) hemi‐epiphytic figs are more susceptible to mortality from soil‐borne fungi than free‐standing figs. As hemi‐epiphytes germinate in the canopy, they potentially escape soil‐borne pathogens at the seed stage. Consequently, relaxed selection on their defensive traits may render them susceptible to soil‐borne fungi; (3) inoculation of seeds with non‐pathogenic fungi previously isolated from seeds would exclude fungal pathogens reducing seed mortality.

## 2 Materials and Methods

### 2.1 Study Site

This study was carried out in Pushpagiri Wildlife Sanctuary (12°37′55″ N, 75°39′1″ E), a seasonal tropical forest in the central Western Ghats, India. The forest spans the elevations from 300 to 1200 m, with a pronounced dry season between November and March (< 100 mm rain per month; annual rainfall 6000 mm) (Pushpagiri Wildlife Sanctuary Management Plan).

### 2.2 Seed Collection and Initial Viability

Fruits were collected from four maternal sources for *Ficus callosa* and from two for *Ficus beddomei* as this fig species showed more staggered fruiting. Ripe fruits were either removed from the tree by hand or collected in mesh traps beneath fruiting branches. The seeds were cleaned by rinsing in tap water, then surface‐sterilized by treating them in 95% ethanol for 10 s, followed by 0.5%–0.7% bleach for 2 min and 70% ethanol for another 2 min. The seeds were then air‐dried under low red: far red irradiance to prevent germination.

The initial viability of fresh seeds was determined using germination trials. Surface‐sterilized seeds were placed in petri‐dishes lined with moist paper towels and sealed with parafilm. For *Ficus callosa*, 15 seeds × 4 replicates = 60 seeds were used to obtain the initial germination score. And for *Ficus beddomei*, 15 seeds × 2 replicates = 30 seeds were used. An average germination score for each species was calculated across the replicates.

### 2.3 Obtaining Fungal Cultures for Inoculation Experiments

For inoculation experiments, we used fungi associated with the seeds of our two fig species. As seeds may acquire endophytic fungi from both the maternal tree environment and soil, we used isolates representative of the two transmission pathways. We isolated fungi from seeds in fresh, ripe fruits while in the tree which we will refer to henceforth as pre‐dispersal fungi that is, fungi associated with seeds prior to seed dispersal or exposure to soil. In addition, we used fungi that colonized seeds after burial in soil, henceforth soil‐borne fungi. Pre‐dispersal fungi were collected from each maternal source. Soil‐borne fungi were obtained following a common garden seed burial experiment conducted as part of a larger study to understand host preferences exhibited by soil‐borne fungi among 12 co‐occurring fig species (list of species in Table S1) including *Ficus beddomei* and *Ficus callosa* used in our study.

In brief, we established five common garden plots (4 m by 4 m) at least 1 km apart across Pushpagiri Wildlife Sanctuary. Plots were located in closed canopy conditions and at least 30 m from any *Ficus* species. *Ficus* seeds from multiple maternal sources per species were pooled together and distributed in sets of 30 into nylon mesh seed bags separated by 40 cm and buried 2 cm deep in the soil. The seeds remained buried for 9 months to facilitate fungal colonization before retrieval. A subset of seeds from each bag was used to set up fungal cultures, which were subsequently identified using Sanger sequencing. A subset of cultures was used for the fungal inoculation experiment.

To obtain fungal cultures for inoculation experiments, seeds prior to and following exposure to soil were surface‐sterilized and placed on a sterile glass slide, crushed with another glass slide, and then transferred into a 2 mL microcentrifuge slant containing malt extract agar. The tubes were kept at room temperature until fungi grew out of the seed fragments. For each fig species, 320 cultures from buried seeds (8 seed bags × 5 common garden locations × 8 seeds per bag) and 60 cultures from fresh seeds (20 seeds × 3 maternal sources) were set up.

Fungal subcultures were used for DNA extraction using the REDExtract‐N‐Amp Plant PCR Kit (Sigma Aldrich Chemicals Private Limited, Bangalore, India) and amplified following manufacturer's instructions (Methods S1). The PCR products were sent to the Sanger sequencing facility at the Centre for Cellular and Molecular Biology (CCMB), Hyderabad, India for DNA sequencing. A cutoff of 97% sequence similarity was used to delineate Operational Taxonomic Units (OTUs). The raw sequences were aligned using the software Sequencher (version 5.4.6 DNA sequence analysis software, Gene Codes Corporation, Ann Arbor, MI USA) and consensus sequences found. These were used to search the NCBI database (version 20 September 2023) for fungal identification.

We chose eight representative fungal taxa, three of which were unique to each *Ficus* species and two that were shared (Table 1; NCBI GenBank accession numbers in Table S2). Two of these fungi were isolated prior to seed dispersal and six after burial in the soil. The selected taxa have been previously shown to belong to genera commonly associated with seeds (Sarmiento et al. 2017; Nelson 2018; Zalamea et al. 2021), supporting our choice of taxa for inoculation experiments.

**TABLE 1:** List of fungal taxa obtained from seeds of *Ficus beddomei* and *Ficus callosa* used in the inoculation experiments.

Fungal taxon	Fungal taxon type	Source
*Alternaria* sp.	Pre‐dispersal	*Ficus beddomei*
*Penicillium sclerotiorum*	Soil‐borne	*Ficus beddomei*
*Fusarium proliferatum*	Soil‐borne	*Ficus beddomei*
*Pseudocercospora* sp.	Pre‐dispersal	*Ficus callosa*
*Aspergillus thermomutatus*	Soil‐borne	*Ficus callosa*
*Colletotrichum* sp.	Soil‐borne	*Ficus callosa*
*Xylaria cubensis*	Soil‐borne	Shared
Sordariomycetes sp.	Soil‐borne	Shared

### 2.4 Inoculation Experiments

#### 2.4.1 Screening of Identified Fungal Taxa as Endophytes or Pathogens

To screen the pathogenicity of the fungal taxa identified above, we conducted an inoculation experiment. Fungal cultures were set up in Malt Extract Agar in 60 mm petri plates. After at least three‐quarters of the plate was covered with mycelium, 20 surface‐sterilized seeds were transferred to each plate and incubated for 2 weeks at room temperature. Plates were stored in the dark to prevent seed germination. A fully factorial design with 8 fungal taxa × 2 seed species × 4 replicate plates was used.

Following incubation, a subset of eight seeds from each plate was placed in a second petri dish lined with moist paper and sealed with parafilm. A second subset of eight seeds was surface‐sterilized and transferred to a separate petri dish. This was done to check whether the presence of the mycelium on the exterior surface of seeds or surface‐sterilization impacted germination. Seeds were kept moist and exposed to a 12‐h day/night light cycle (natural light + fluorescent lamp) over 2 months while germination was monitored. Finally, the remaining four seeds from each plate were set up for fungal culture, followed by DNA extraction and sequencing to confirm successful fungal inoculation (Koch's postulates).

The final germination scores after incubation in fungal cultures were normalized with respect to the initial viability of seeds (determined in Section 2.2), that is, proportion of seeds germinated after incubation in fungal taxon/proportion of fresh seeds germinated. The fungi in which the final normalized germination score was very low were designated as pathogens; others with no detectable reduction in germination or less negative effects than the pathogens were designated as endophytes.

#### 2.4.2 Testing the Effect of Endophytes Against Pathogens

To test whether endophytes can reduce pathogen induced mortality of seeds, seeds were incubated with an endophyte followed by a pathogen and then set up for germination. A total of 5 endophytes × 2 pathogens × 4 replicates were set up for *Ficus callosa*. For *Ficus beddomei*, 2 endophytes × 2 pathogens × 4 replicates were set up. The number of endophytes tested for each species was based on results of the screening experiment (Section 3.1). In total, 35 surface‐sterilized seeds were placed in each endophyte plate. Seeds were incubated with endophytes in a dark cabinet for 10 days at room temperature, then transferred to the pathogen plate and incubated for another 10 days. Seeds were then set up for germination (15 seeds surface‐sterilized and 15 without surface‐sterilization). The remaining five seeds from each plate were transferred for fungal culturing to assess whether fungal inoculation was successful.

The final germination scores were normalized with respect to the initial germination score of fresh seeds. These were compared against controls where seeds were incubated only with pathogens (obtained from the screening experiment) normalized with respect to fresh seeds.

### 2.5 Statistical Analyses

To test whether the pathogenicity of fungal taxa differed within and among tree species (first inoculation experiment), we used a linear model (model M1) with germination score as response variable and fungal taxon, tree species and their interactions as explanatory variables (R version 4.4.3, base package). The model formula is given below:

$$\frac{\left(\text{Germination in fungal taxon}\right)}{\left(\text{Germination of fresh seeds}\right)}\sim \text{Tree species}\times \text{Fungal taxon}$$

Here, tree species include *Ficus beddomei* and *Ficus callosa*. Fungal taxon values include no fungal inoculation or control, *F*. *proliferatum*, *P*. *sclerotiorum*, *Alternaria* sp., *Pseudocercospora* sp., *A. thermomutatus*, *Colletotrichum* sp., Sordariomycetes sp., and *X. cubensis*.

We used the functions *emmeans* and *contrast* (R package *emmeans*) with “tukey” adjustments to obtain pairwise comparisons of all the fungal taxa against “No fungal inoculation” within each tree species.

To test whether exposure to endophyte prior to pathogen influenced seed survival (the serial endophyte‐pathogen experiment), we analyzed germination scores with tree species, endophyte taxon, pathogen taxon, and their interactions as explanatory variables (model M2):

$$\begin{array}{c} \frac{\left(\text{Germination after incubation in endophyte followed}\;\mathrm{by}\;\text{pathogen}\right)}{\left(\text{Germination of fresh seeds}\right)}\\ \sim \text{Tree species}\times \text{Endophyte}\times \text{Pathogen} \end{array}$$

Here, tree species include *Ficus beddomei* and *Ficus callosa*. Endophyte values include control or no endophyte, *Alternaria* sp., *Pseudocercospora* sp., *A. thermomutatus*, *Colletotrichum* sp., and Sordariomycetes sp., and Pathogen values include *F*. *proliferatum* and *P*. *sclerotiorum*.

The functions *emmeans* and *contrast* (R package *emmeans*) with ‘tukey’ adjustments were used to obtain pairwise comparisons of all the endophytes against “No endophyte” within each category grouped by tree species and pathogen.

## 3 Results

The initial viability of seeds prior to the inoculation experiments was found to be 100% for *Ficus callosa* and 63% ± 16% (mean ± SE) for *Ficus beddomei*, respectively.

### 3.1 Isolation and Characterization of Seed‐Associated Fungi

We found that fungi took from one week to over a year to emerge in the cultures. Isolates were obtained from 5%–10% of fresh (unburied) seeds, and 45%–54% for buried seeds. In total, 71 cultures were processed from buried seeds, 37 from *Ficus callosa* seeds and 34 from *Ficus beddomei* seeds. In addition, 3 cultures were processed from fresh seeds of each tree species.

### 3.2 Pathogenicity of Pre‐Dispersal and Soil‐Borne Fungal Taxa

**FIGURE 1:**
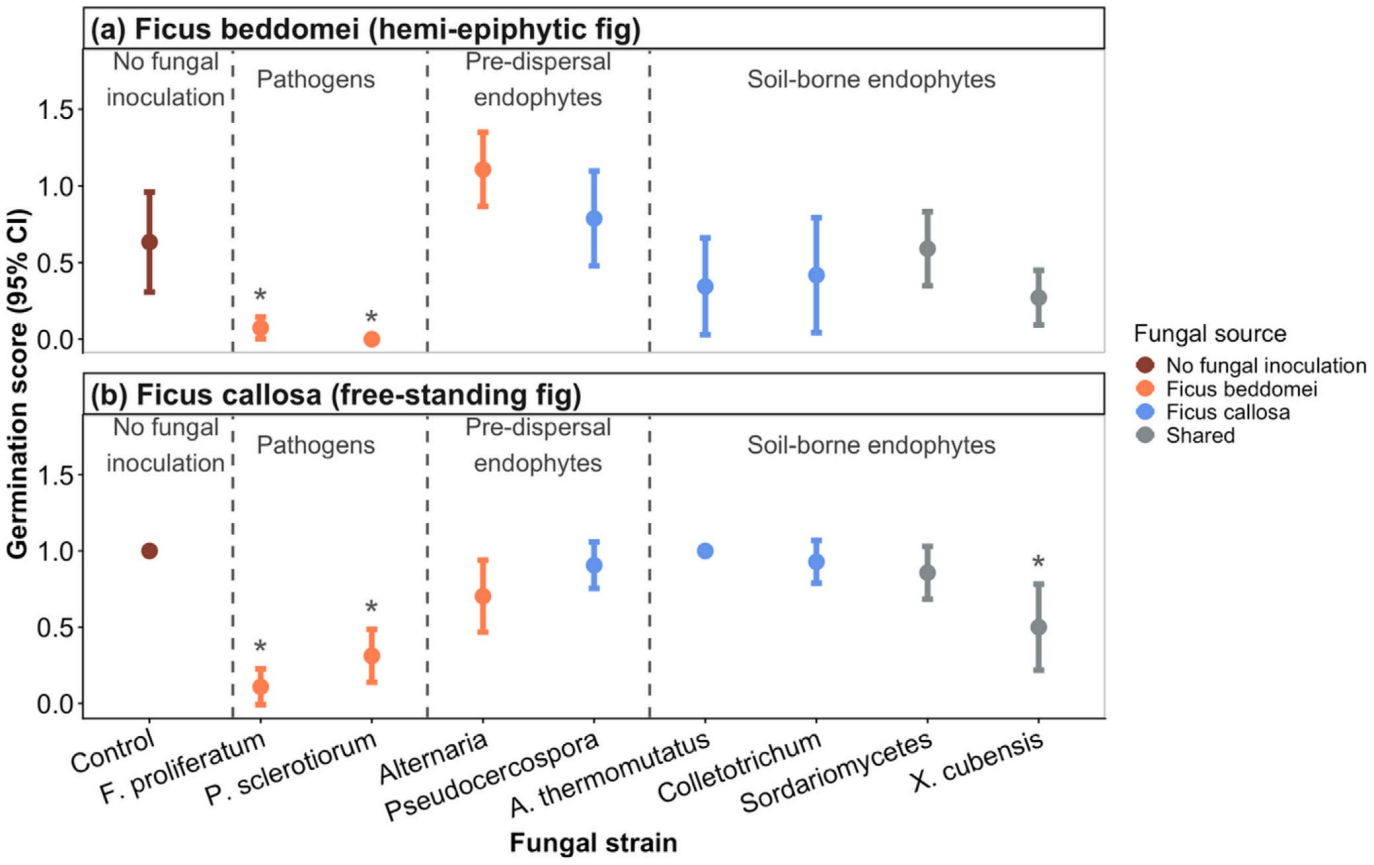
Germination scores for inoculation treatments scaled with respect to initial viability of seeds that is, germination with no fungal inoculation for the screening of fungal taxa for pathogenicity. Seeds of (a) *Ficus beddomei* and (b) *Ficus callosa* were inoculated with select fungal taxa in the x‐axis. Fungal taxa in which germination was significantly different from the control (no fungal inoculation) are indicated by asterisks. *F. proliferatum* and *P. sclerotiorum* were designated as pathogens as these two taxa substantially reduced seed germination compared to initial viability. Fungal taxa were designated as endophytes if they had a less negative effect on germination than *F. proliferatum* or *P. sclerotiorum*.

Eight seed‐fungal taxon combinations were tested for each fig species (Figure 1a,b). These taxa were classified into three categories based on their effects on seed germination. Two taxa were designated as ‘pathogens’ that significantly reduced germination relative to no fungal inoculation, while two other groups, ‘pre‐dispersal endophytes’ and ‘soil‐borne endophytes’ (depending on source of fungal taxa), had either little effect on germination relative to seeds with no fungal inoculation or less negative effects than pathogens. Across both fig species, *F*. *proliferatum* and *P*. *sclerotiorum* reduced germination by 91% and 85% respectively compared to no inoculation. For the hemi‐epiphyte *Ficus beddomei*, seeds that were inoculated with the two pre‐dispersal endophytes (*Alternaria* sp. and *Pseudocercospora* sp.) showed similar germination rate as seeds with no fungal inoculation, whereas germination in three out of four soil‐borne endophytes was intermediate between initial viability and pathogen inoculation (Figure 1a; Table 2). In contrast, for the free‐standing fig, *Ficus callosa*, germination was comparable to the no inoculation control for both pre‐dispersal and soil‐borne fungal taxa designated as ‘endophytes’ (except *X. cubensis*, Figure 1b; Table 2).

**TABLE 2:** First inoculation experiment involving screening of pathogenicity of fungi: Results of the linear model M1: (Germination in fungal taxon)/(Germination of fresh seeds) ~ Tree species × Fungal taxon showing pairwise comparisons against designated pathogens *Fusarium proliferatum* and *Penicillium sclerotiorum* that are significant.

Tree species	Pairwise comparison of fungal taxa	Effect size	*p*
*Ficus beddomei*	*F. proliferatum* – No fungal inoculation	−0.55 ± 0.24	0.08
*Ficus beddomei*	*P. sclerotiorum* – No fungal inoculation	−0.63 ± 0.24	0.07
*Ficus beddomei*	*Alternaria* sp. – *F. proliferatum*	1.03 ± 0.15	< 0.001
*Ficus beddomei*	*Alternaria* sp. – *P. sclerotiorum*	1.1 ± 0.15	< 0.001
*Ficus beddomei*	*Pseudocercospora* sp. – *F. proliferatum*	0.71 ± 0.15	0.001
*Ficus beddomei*	*Pseudocercospora* sp. – *P. sclerotiorum*	0.79 ± 0.15	< 0.001
*Ficus beddomei*	Sordariomycetes sp. – *F. proliferatum*	0.52 ± 0.15	0.008
*Ficus beddomei*	Sordariomycetes sp. – *P. sclerotiorum*	0.59 ± 0.15	0.0016
*Ficus callosa*	*F. proliferatum* – No fungal inoculation	−0.89 ± 0.19	< 0.001
*Ficus callosa*	*P. sclerotiorum* – No fungal inoculation	−0.69 ± 0.19	0.0031
*Ficus callosa*	*X*. *cubensis* – No fungal inoculation	−0.50 ± 0.19	0.07
*Ficus callosa*	*Alternaria* sp. – *F. proliferatum*	0.59 ± 0.15	0.02
*Ficus callosa*	*Alternaria* sp. – *P. sclerotiorum*	0.39 ± 0.15	0.09
*Ficus callosa*	*Pseudocercospora* sp. – *F. proliferatum*	0.79 ± 0.15	< 0.001
*Ficus callosa*	*Pseudocercospora* sp. – *P. sclerotiorum*	0.59 ± 0.15	0.02
*Ficus callosa*	*A. thermomutatus* – *F. proliferatum*	0.89 ± 0.15	< 0.001
*Ficus callosa*	*A. thermomutatus* – *P. sclerotiorum*	0.69 ± 0.16	0.004
*Ficus callosa*	*Colletotrichum* sp. – *F. proliferatum*	0.82 ± 0.16	< 0.001
*Ficus callosa*	*Colletotrichum* sp. – *P. sclerotiorum*	0.62 ± 0.16	0.02
*Ficus callosa*	Sordariomycetes sp. – *F. proliferatum*	0.74 ± 0.16	0.001
*Ficus callosa*	Sordariomycetes sp. – *P. sclerotiorum*	0.54 ± 0.16	0.007
*Ficus callosa*	*X*. *cubensis* – *F. proliferatum*	0.39 ± 0.15	0.09

*Note:* A positive effect size indicates that the first fungal taxon exhibits higher germination than the second and vice‐versa, with the magnitude of the effect representing the percentage difference between them.

The effect of fungal inoculation on germination differed between the two fig species (Tree species × Fungal taxon interaction, *p* < 0.001). *Ficus beddomei* showed significantly lower germination than *Ficus callosa* for three out of four soil‐borne fungi, namely, *A. thermomutatus*, *Colletotrichum* sp., and Sordariomycetes sp. (Table 3). We found that surface‐sterilization after inoculation with fungal taxon did not affect germination. Based on these results, the pre‐dispersal endophytes, *Alternaria* sp. and *Pseudocercospora* sp., were chosen for serial inoculation experiments using the hemi‐epiphytic fig. These same fungal taxa, along with the soil‐borne endophytes, *A. thermomutatus*, *Colletotrichum* sp. and Sordariomycetes sp. were used for the serial inoculation experiments using the free‐standing fig.

**TABLE 3:** Inoculation experiment to screen pathogenicity of fungi: Results of the linear model M1: (Germination in fungal taxon)/(Germination of fresh seeds) ~ Tree species × Fungal taxon showing pairwise comparisons between the two tree species inoculated with different fungal taxa.

Fungal taxon	Pairwise comparison of tree species	Effect size	*p*
*Alternaria* sp.	*Ficus callosa* – *Ficus beddomei*	−0.40 ± 0.15	0.01
*A. thermomutatus*	*Ficus callosa – Ficus beddomei*	0.66 ± 0.15	< 0.001
*Colletotrichum* sp.	*Ficus callosa* – *Ficus beddomei*	0.51 ± 0.15	0.0017
*P. sclerotiorum*	*Ficus callosa* – *Ficus beddomei*	0.31 ± 0.15	0.04
Sordariomycetes sp.	*Ficus callosa* – *Ficus beddomei*	0.27 ± 0.16	0.09

*Note:* A positive effect size means *Ficus callosa* has higher germination than *Ficus beddomei* in the given fungal taxon and vice‐versa, with the magnitude representing the percentage difference in germination between them.

### 3.3 Effects of Serial Inoculation of Seeds With Endophytes and Pathogens

**FIGURE 2:**
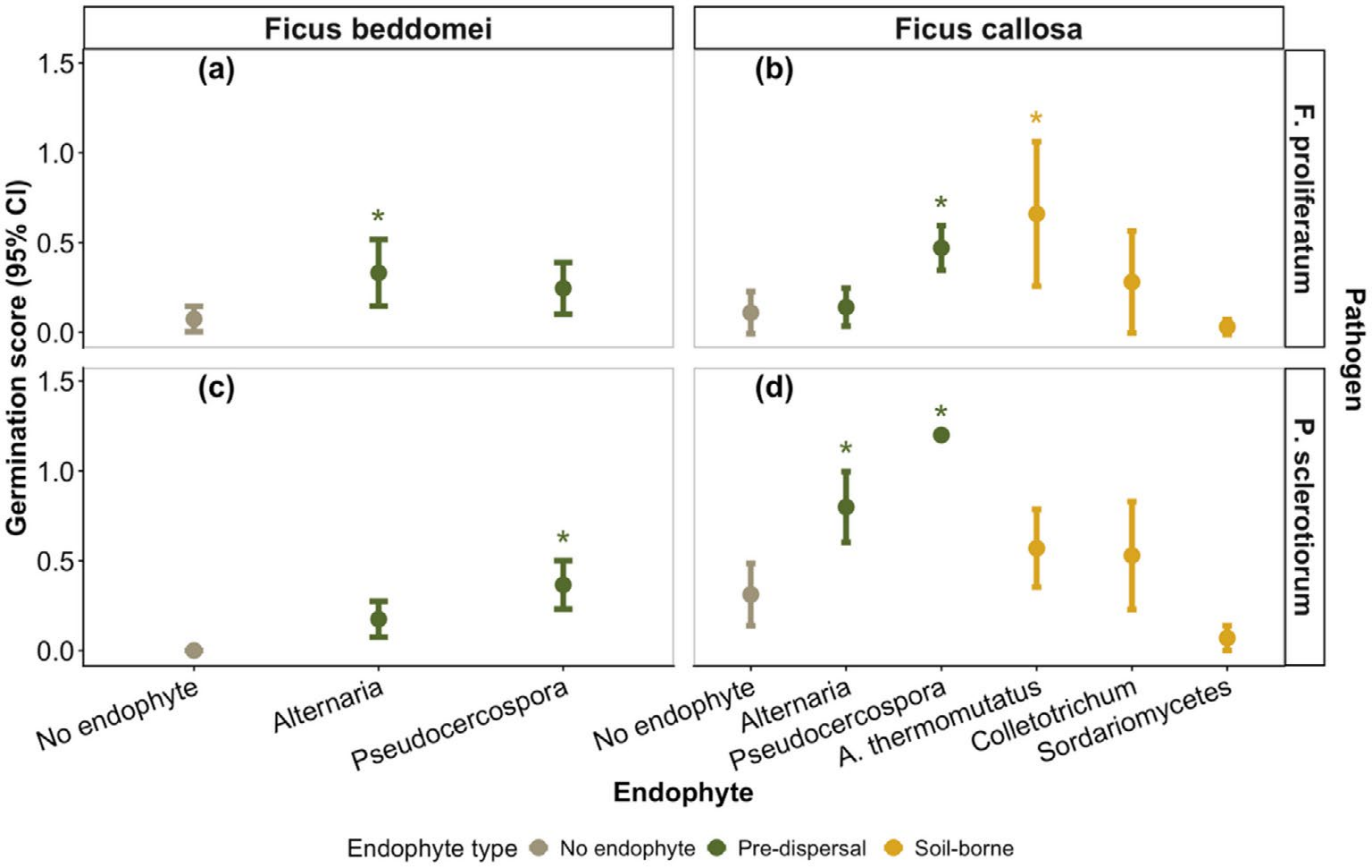
Germination scores on the y‐axis normalized with respect to initial viability after successive inoculation of seeds with an endophyte followed by a pathogen (endophytes indicated on the x‐axis and pathogens, *F. proliferatum* and *P. sclerotiorum* on the rows of the panels). Asterisks indicate endophyte‐pathogen combinations that resulted in significantly higher germination as compared to endophyte‐free controls (first bar in each plot) for *Ficus beddomei* (a, c) and *Ficus callosa* (b, d).

The three‐way interaction between tree species, endophyte, and pathogen was not significant indicating that the combined effects of endophytes and pathogens were broadly consistent between the tree species. Nevertheless, significant two‐way interactions were observed (tree species × endophyte, tree species × pathogen, endophyte × pathogen), reflecting tree species‐specific and pathogen‐specific responses. Out of the 14 seed‐endophyte‐pathogen combinations tested, six showed significantly higher germination when seeds were treated with an endophyte prior to the pathogen as compared to the endophyte‐free controls. Four of these involved the free‐standing fig, *Ficus callosa*, and included both pre‐dispersal and soil‐borne endophytes. For the hemi‐epiphyte, *Ficus beddomei*, one pre‐dispersal endophyte (*Alternaria* sp.) significantly enhanced germination in the presence of the pathogen *P*. *sclerotiorum* (Figure 2a,c, Table 4), while another (*Pseudocercospora* sp.) had a positive effect in the presence of *F*. *proliferatum*. However, the magnitude of these effects was smaller than that observed for the free‐standing fig. For *Ficus callosa*, the effects of the endophytes were observed against both the pathogens *F*. *proliferatum* and *P*. *sclerotiorum* (Figure 2b,d, Table 4). Additionally, the endophyte *Pseudocercospora* sp. showed a stronger effect against *P*. *sclerotiorum*, improving germination to a greater degree than against *F*. *proliferatum*. There was no effect of surface‐sterilization after inoculation of endophytes and pathogens on germination.

**TABLE 4:** Serial inoculation experiment involving exposure of seeds to endophyte followed by pathogen: Results of the linear model M2: (Germination after incubation in endophyte followed by pathogen)/(Germination of fresh seeds) ~ Tree species × Endophyte × Pathogen, showing significant pairwise comparisons of endophyte + pathogen combinations against respective controls (no endophyte).

Tree species	Comparison of endophyte‐pathogen combination against control (no endophyte)	Effect size	*p*
*Ficus callosa*	*A. thermomutatus + F. proliferatum*	0.55 ± 0.15	0.002
*Ficus callosa*	*Pseudocercospora* sp. *+ F. proliferatum*	0.36 ± 0.15	0.04
*Ficus callosa*	*Alternaria* sp. + *P. sclerotiorum*	0.49 ± 0.15	0.003
*Ficus callosa*	*Pseudocercospora* sp. + *P. sclerotiorum*	0.89 ± 0.15	< 0.0001
*Ficus beddomei*	*Alternaria* sp. + *F. proliferatum*	0.25 ± 0.08	0.01
*Ficus beddomei*	*Pseudocercospora* sp. + *P. sclerotiorum*	0.37 ± 0.08	0.0003

*Note:* A positive effect size indicates that the endophyte + pathogen treatment exhibits higher germination than the control (no endophyte), with the magnitude of the effect representing the percentage difference between them.

## 4 Discussion

Soil‐borne fungal and oomycete pathogens cause substantial mortality of seeds and seedlings in tropical forests (Bell et al. 2006; Bagchi et al. 2010). Seeds of pioneer trees which constitute the bulk of seeds in the soil seed bank are highly vulnerable to these fungi (Dalling et al. 1998). However, not all seed‐fungal interactions are necessarily lethal. We tested a mechanism by which seeds of figs, a group of important pioneer trees, could defend themselves by acquiring beneficial endophytic fungi either from the maternal tree environment (pre‐dispersal endophytes) or from the soil. We found some support for microbially‐mediated seed defense in fig seeds using ex‐situ inoculation experiments highlighting the potential for non‐lethal seed‐fungal interactions in tropical forest soil to shape seed survival.

### 4.1 Pathogenicity of Pre‐Dispersal and Soil‐Borne Fungi

We selected a suite of pre‐dispersal and soil‐borne fungi to test their effects on seed survival. We found support for our first hypothesis—the pre‐dispersal fungi had non‐lethal effects on both fig species. In contrast, the two fungi found to be pathogenic, *F. proliferatum* and *P. sclerotiorum*, were both isolated from the hemi‐epiphytic fig after seed‐burial in the soil. The effects of other soil‐borne fungal taxa on seed survival varied between the fig species. This is consistent with the findings of Sarmiento et al. (2017) who found that a fungal taxon can have a negative effect on seed survival on one host while being asymptomatic in another. Our second hypothesis, that the hemi‐epiphytic fig would have reduced defenses against seed pathogens, was also supported, with significantly lower germination for three out of the remaining four soil‐borne fungal taxa.

### 4.2 Priority Effects as a Mechanism of Seed Defense

Previous experimental work has shown that endophytes can exclude pathogens from leaves to reduce disease severity (Arnold et al. 2003; Leopold and Busby 2020). Similar priority effects have been observed in the colonization of root tips by ectomycorrhizal fungi and dark‐septate endophytes and exclusion of pathogens (Kennedy et al. 2009; Ridout et al. 2019). Our serial inoculation experiment suggests that priority effects could be a plausible mechanism of seed defense. For the free‐standing fig, initial infection with an endophyte resulted in higher germination after subsequent exposure to a pathogen as compared to pathogen‐only controls, providing support to our third hypothesis on priority effects of endophytes. However, the effect of the endophyte depended on the pathogen. The soil‐borne endophyte, *A. thermomutatus* reduced seed mortality when exposed to the pathogen *F*. *proliferatum*, while the pre‐dispersal endophyte *Alternaria* sp. reduced seed mortality when exposed to the pathogen *P*. *sclerotiorum*. Another pre‐dispersal endophyte, *Pseudocercospora* sp., showed a higher effect against *P*. *sclerotiorum* than *F*. *proliferatum*. Therefore, an endophyte that excludes one pathogen may not be effective against others.

This finding of the context‐dependency of endophyte‐pathogen interactions has implications for seed mortality due to fungal pathogens. First, it suggests that the virulence of fungal pathogens that seeds encounter in the soil may depend on the prior endophytic infection status of seeds. This could potentially contribute to the variation in mortality rates across host species and even among individuals within the same species in this study, and as observed by Sarmiento et al. (2017) when inoculating seeds with the same pathogen. Second, a diversity of endophytes in seeds may provide host biotic resistance to seed losses to pathogens in soil seed banks. The mechanisms by which endophytes confer protection against lethal pathogen infection in seeds remain unclear. One possibility could be that the endophyte may produce secondary metabolites that confer disease resistance against pathogens (Schulz et al. 1999). Or endophytes could induce chemical defenses in seeds via action of enzymes that selectively affect pathogens (Fuerst et al. 2014, 2018).

### 4.3 Weaker Priority Effects in the Hemi‐Epiphytic Fig

We found that the free‐standing fig could benefit from several non‐lethal associations with pre‐dispersal and soil‐borne fungi for defense against pathogens. For the hemi‐epiphyte, the effect of endophytes was smaller when compared to the free‐standing fig. This suggests that pathogen exclusion by endophytes is likely to be beneficial for the free‐standing fig that recruits from the soil. Our inferences are limited as only one species from each habit type was tested. However, a larger experiment using 12 fig species (5 free‐standing figs and 7 hemi‐epiphytes) showed that the hemi‐epiphytes suffered greater mortality after 9 months of burial in the soil than free‐standing figs (Sundararajan et al. unpublished data).

For the hemi‐epiphytic fig, the weaker effect of the endophytes may be attributed to a few factors. Terrestrial and arboreal soils have been previously demonstrated to have substantial differences in fungal communities (Gora et al. 2019). We did not place seeds in the canopy and therefore could not characterize post‐dispersal infections in this microsite. It is possible that the pre‐dispersal endophytes we cultured from the hemi‐epiphytic fig might have stronger priority effects against pathogens in arboreal soil, or that post‐dispersal endophytes from arboreal soil could aid in better seed defense for the hemi‐epiphyte. Additionally, the epiphytic lifestyle has been proposed as an evolutionary escape from soil‐borne enemies (Spicer et al. 2020). It would follow that there will be reduced selection on the defensive traits of epiphytes making them vulnerable to fungi from terrestrial soil. Moreover, previous work has shown that plant defense could be hierarchical with genetic resistance in plants contributing more than priority effects due to endophytes against pathogens (Busby et al. 2019). Therefore, future work exploring the diversity of genes for pathogen resistance in free‐standing and hemi‐epiphytic figs would provide a better understanding of defensive capacity between the two habits.

The simplified microbial community of seeds compared to other plant tissues makes them particularly attractive for experiments to unravel seed‐fungal interactions. Yet, inoculation experiments have some limitations restricting our inferences about how microbially mediated seed defense will play out in the forest. Soil fungal communities are inherently complex, and interspecific interactions among fungi are likely to be important within the host (Kennedy et al. 2009). Inoculation with a single fungal isolate may not replicate these interactions. Moreover, incubation duration may also influence the outcome of these interactions (Nelson and Hauser 2025). Nevertheless, understanding pairwise interactions between host and fungus, and endophyte and pathogen is a crucial first step. The next steps to implementing an experiment in the field could be to first assess the presence of symbionts in individual seeds and then transfer seeds to the soil thereby capturing interactions with the soil fungal community. The importance of primary infection including its retention and the extent of regulation of fungal communities by interspecific interactions among co‐infecting fungi within the host are key remaining questions.

Finally, the question arises of transmission of beneficial fungi from one generation to another. While vertical transmission of fungal endophytes has been well established in grasses (Saikkonen et al. 2010; Perez et al. 2017), there is limited evidence for vertical transmission of fungi in tropical trees (Bayman et al. 1998). Developing functionally specific associations with horizontally transmitted fungi could add to a plant's defensive repertoire, which could potentially contribute to long term seed persistence in the soil seed bank. By positively affecting seed survival in the presence of fungal pathogens, endophyte mediated seed defenses could have important consequences for how density or distance‐dependent mortality unfolds among seeds. It would be insightful to test whether these endophytes are retained in seedling tissue, and their impacts on seedling growth or defense (Bergna et al. 2018; Abdelfattah et al. 2023). Understanding the role of endophytes in plant regeneration will elucidate the depth of these symbioses that seem to arise dynamically between plants and a diverse array of tropical endophytic fungi.

## Author Contributions

**Sumashini Sundararajan:** conceptualization (lead), data curation (equal), formal analysis (lead), funding acquisition (lead), investigation (supporting), methodology (lead), project administration (lead), writing – original draft (lead), writing – review and editing (equal). **Gauranshi Chamoli:** data curation (equal), investigation (lead), methodology (equal). **James W. Dalling:** conceptualization (equal), funding acquisition (equal), methodology (equal), writing – review and editing (equal). **Meghna Krishnadas:** conceptualization (equal), methodology (equal), resources (lead), writing – review and editing (equal).

## Funding

This work was supported by American Philosophical Society, Lewis and Clark Fund 2022, Arnold Arboretum, Harvard Ashton Award 2023.

## Disclosure

Statement on Inclusion: Our study brings together authors from USA (J.W.D.) and India (S.S., G.C. and M.K.). It contributed to doctoral training of one Indian student (SS), capacity building for G.C. in scientific techniques, and is a part of a larger research project involving US and Indian scientists.

## Conflicts of Interest

The authors declare no conflicts of interest.

## Supporting information


**Table S1:** List of *Ficus* species included in the common garden experiment.
**Methods S1**. Molecular characterization of fungi from cultures: DNA extraction and amplification.
**Table S2:** List of fungal taxa used in the inoculation experiments along with their GenBank accession numbers.
**Table S2:** List of fungal taxa used in the inoculation experiments along with their GenBank accession numbers.

## Data Availability

Sequences used in this study are deposited in NCBI GenBank under accession numbers: PX498363–PX498366 and PX498368–PX498371. Germination data has been deposited in Illinois Data Bank under https://doi.org/10.13012/B2IDB‐6683417_V1.
