# Supplementary material for: Getting by With a Little Help From Fungal Friends: Seed‐Associated Endophytes Contribute to Seed Defense Against Fungal Pathogens in Keystone Tropical Trees
Source: Ecol Evol. 2026 Aug 12;16(8):e74184. doi: 10.1002/ece3.74184 (PMC13469350; doi:10.1002/ece3.74184)
Supplement: Supplementary file 1 [file ECE3-16-e74184-s001.docx]

**Supporting Information**

**Getting by with a little help from fungi friends: seed endophytes contribute to seed defense against fungal pathogens in keystone tropical trees**

Sumashini Sundararajan, Gauranshi Chamoli, James W Dalling, Meghna Krishnadas

**Table S1**: List of *Ficus* species included in the common garden experiment

| **S.no** | **Free-standing *Ficus* species** | **Hemi-epiphytic *Ficus* species** |
| --- | --- | --- |
| 1. | *Ficus nervosa* | *Ficus beddomei* |
| 2. | *Ficus callosa* | *Ficus amplissima* |
| 3. | *Ficus arnottiana* | *Ficus talbotii* |
| 4. | *Ficus hispida* | *Ficus tsjahela* |
| 5. | *Ficus racemosa* | *Ficus microcarpa* |
| 6. |  | *Ficus travancorica* |
| 7. |  | *Ficus virens* |
| 8. |  | *Ficus altissima* |

**Methods S1. Molecular characterization of fungi from cultures: DNA extraction and amplification**

Fungi were isolated from surface-sterilized seeds placed on 2% malt extract agar medium in 2 ml microcentrifuge tubes (see main text section 2.3 for setting up of fungal cultures). We used the REDExtract-N-Amp Plant PCR kit (Sigma-Aldrich Chemicals Private Limited, Bangalore, India) for DNA extraction and amplification by PCR following a modified protocol for fungal mycelium (1). Under a biological safety cabinet, a small piece of mycelium was extracted from each culture using a sterile pipette tip and transferred to a 1.5 ml microcentrifuge tube containing 100 µL of the extraction solution (part of the kit). The piece of mycelium was macerated in the extraction solution using the pipette tip. The tube containing mycelium and extraction solution was then incubated in a heat block at 95°C for 10 minutes, and then 100 µL of dilution solution (part of the kit) was added (1). Primers ITS1F and LR3 were used to amplify using PCR a region spanning the internal transcribed spacers and 5.8S gene (ITS rDNA) and a portion of the large subunit (LSU rDNA), constituting a total length of 1000-1200 base-pair (bp) fragment. Each reaction mixture included 10 µL of the Sigma Aldrich REDExtract-N-Amp PCR ReadyMix, 0.8 µL of each primer, 6.4 µL of PCR-quality water and 2 µL of DNA template. Cycling reactions (95ºC for 3 min; 36 cycles of 95ºC for 30s, 54ºC for 30s, 72ºC for 1 min; and 72º C for 10 min) were run on a thermocycler.

PCR products were stained with SYBR Green 1 (Invitrogen, CA, USA) followed by electrophoresis on a 1% agarose gel. All the samples that yielded a band of the expected size (~1200 bp), were submitted to the Sanger sequencing facility at the Center for Cellular and Molecular Biology, Hyderabad, India.

**Table S2:** List of fungal taxa used in the inoculation experiments along with their GenBank accession numbers

| **Fungal taxon** | **Fungal taxon type** | **Source** | **GenBank accession number** |
| --- | --- | --- | --- |
| *Alternaria* sp. | pre-dispersal | *Ficus beddomei* | [PX498363](https://www.ncbi.nlm.nih.gov/nuccore/PX498363) |
| *Penicillium sclerotiorum* | soil-borne | *Ficus beddomei* | [PX498364](https://www.ncbi.nlm.nih.gov/nuccore/PX498364) |
| *Fusarium proliferatum* | soil-borne | *Ficus beddomei* | [PX498365](https://www.ncbi.nlm.nih.gov/nuccore/PX498365) |
| *Pseudocercospora* sp. | pre-dispersal | *Ficus callosa* | [PX498366](https://www.ncbi.nlm.nih.gov/nuccore/PX498366) |
| *Aspergillus thermomutatus* | soil-borne | *Ficus callosa* | [PX498368](https://www.ncbi.nlm.nih.gov/nuccore/PX498368) |
| *Colletotrichum* sp. | soil-borne | *Ficus callosa* | [PX498369](https://www.ncbi.nlm.nih.gov/nuccore/PX498369) |
| *Xylaria cubensis* | soil-borne | shared | [PX498370](https://www.ncbi.nlm.nih.gov/nuccore/PX498370) |
| Sordariomycetes sp. | soil-borne | shared | [PX498371](https://www.ncbi.nlm.nih.gov/nuccore/PX498371) |

**References**

1. https://www.sigmaaldrich.com/US/en/technical-documents/technical-article/genomics/dna-and-rna-purification/extract-n-amp-plant-kits
